# Panel Doctors and Prescription Writing

**Published:** 1913-09-13

**Authors:** 


*To the Editor of* The Hospital.

### Panel Doctors and Prescription Writing.

*To the Editor of* The Hospital.

Sir,—I should be much obliged if you could clear up for me a small point in connection with the working of the Insurance Act, about which your frequent articles are so illuminating and exhaustive. I am not a panel practitioner myself, and a few days ago I was attending a householder in the ordinary way of private practice when he asked me to look at one of his servants who was in bed and complaining of illness. I found the servant had a high temperature, rapid pulse, and sore throat, whereupon the employer suggested that the woman's panel doctor should be sent for. I assented to this, of course, and did not see the woman again professionally. The panel doctor was telephoned for, and came within a reasonable time—about two hours. He agreed in the diagnosis of severe tonsillitis, and ordered some quite sensible treatment. He insisted, however, that to obtain the necessary medicines, gargle, and so on, it was necessary for a messenger to go to his surgery, more than a mile distant, and to take the prescriptions thence to a chemist to be made up. He stated that the regulations did not allow him to write the prescriptions on the spot.

I feel quite sure that no such absurd regulation can exist, and that the practitioner was either mistaken or attempting to discourage his patients from sending for him. But I should be glad to know whether this is so or not.

Furthermore, when the patient was barely convalescent, that is to say, just out of bed and able to walk across the room, he insisted that she should present herself the next morning at his surgery, distant as aforesaid, at (I think) nine o'clock. I can, if necessary, supply in strict confidence the name of the doctor (he is on the London panel), but I take it you will be able to pronounce upon the facts of the case without this. Thanking you in anticipation.—Yours truly,

Medicus.

[The regulations require that prescriptions shall be furnished on printed forms. The panel doctor has also to keep a note of the prescription and treatment given. If, therefore, he did not have a prescription form with him when he called upon the patient, a messenger could be sent to his surgery as the most expeditious way for the medicine to be obtained. Were it not that instances have been known to the contrary, it would have to be presumed that the doctor considered the patient capable of attending his surgery for treatment, otherwise he would not have required her to make the journey. But, unfortunately, the logic of facts makes our correspondent's contention just as likely to be true. The hours of attendance are fixed by arrangement with the local Insurance Committee. —Ed. The Hospital.]

